# Verification of Budesonide/Glycopyrronium/Formoterol (BGF) Triple Therapy in the Department of Cardiology: A Pilot Study

**DOI:** 10.7759/cureus.110204

**Published:** 2026-06-03

**Authors:** Takahiro Kamihara, Shinji Kaneko, Takuya Omura, Toyoaki Murohara, Atsuya Shimizu

**Affiliations:** 1 Department of Cardiology, National Center for Geriatrics and Gerontology, Obu, JPN; 2 Department of Cardiology, Toyota Kosei Hospital, Toyota, JPN; 3 Department of Diabetes and Endocrinology, National Center for Geriatrics and Gerontology, Obu, JPN

**Keywords:** bgf triple therapy, body mass index (bmi), cardiology, copd (chronic obstructive pulmonary disease), heart failure

## Abstract

Background

Chronic obstructive pulmonary disease (COPD) frequently coexists with heart failure (HF), worsening patient prognosis. While single-inhaler triple therapy (BGF: budesonide/glycopyrronium/formoterol) is effective, the clinical impact of its initiation by cardiologists, who often manage latent COPD in patients with HF, remains underexplored. This study aimed to evaluate the validity and safety of BGF therapy managed by cardiologists compared to respiratory specialists and to identify prognostic factors associated with acute exacerbations.

Methods

This retrospective pilot study analyzed 22 outpatients who received BGF therapy between April 2024 and March 2025. Outcomes were compared between the Department of Cardiology and the Department of Pulmonary Medicine. Statistical analyses included Kaplan-Meier survival curves and Cox proportional hazards regression.

Results

No significant differences were found in mortality, hospitalization, or exacerbation rates between departments. The cardiology group had a significantly higher prevalence of HF and more frequent treatment initiation. Multivariate analysis revealed that comorbid HF significantly increased the risk of acute exacerbations (hazard ratio (HR) > 1), while higher body mass index was a protective factor (obesity paradox).

Conclusions

BGF triple therapy is safe and effective under cardiologist management. Proactive intervention is particularly justified in patients with HF, where the risk of exacerbation remains high regardless of the severity of the primary respiratory symptom.

## Introduction

Chronic obstructive pulmonary disease (COPD) is one of the leading causes of mortality worldwide [1], and inhalation therapy plays a critical role in its management [2]. Recently, single-inhaler triple therapy (BGF: budesonide/glycopyrronium/formoterol) - combining an inhaled corticosteroid (ICS), a long-acting muscarinic antagonist (LAMA), and a long-acting beta 2-agonist (LABA) - has emerged, demonstrating high efficacy in reducing acute exacerbations and improving lung function [3-5].

It is well-established that patients with COPD frequently present with cardiovascular comorbidities, particularly heart failure [6], and these conditions mutually exacerbate the prognosis [7-9]. In clinical practice, COPD management is primarily conducted in respiratory departments. However, a significant number of undiagnosed or untreated COPD cases may be latent among patients visiting cardiology departments primarily for heart failure (HF) management. Nevertheless, there is insufficient evidence regarding the clinical impact of cardiologist-led initiation and maintenance of BGF triple therapy, as well as its efficacy and safety compared with management by respiratory specialists.

This study aimed to provide a preliminary evaluation of a collaborative care model. The primary objective of this pilot study was to assess the clinical feasibility and safety of single-inhaler BGF triple therapy when initiated and managed by cardiologists compared with pulmonologists in patients with comorbid COPD and HF. The secondary objective was to explore potential baseline prognostic factors associated with acute exacerbations of COPD within this combined cohort. We hypothesized that under strict cardiovascular monitoring, cardiologist-led management of BGF therapy would be safe and feasible, serving as a hypothesis-generating foundation for future multicenter trials.

## Materials and methods

This study was conducted with the approval of the Ethics and Conflict of Interest Committee of the National Center for Geriatrics and Gerontology (approval no. 1761-3), in accordance with the Declaration of Helsinki. The Ethics and Conflict of Interest Committee of the National Center for Geriatrics and Gerontology waived the requirement for participant consent and approved the use of an opt-out informed consent method for this study.

This retrospective pilot study included 22 outpatients who received BGF triple therapy between April 1, 2024, and March 31, 2025. Clinical data were collected from electronic medical records to verify the impact of BGF therapy administered within the Department of Cardiology.

COPD was diagnosed based on a history of smoking and spirometry results showing a forced expiratory volume in 1 second to forced vital capacity ratio (FEV1/FVC) of less than 0.7. Patients diagnosed with COPD and prescribed BGF were included. Acute exacerbation was defined as a state in which a physician determined that respiratory distress, cough, and sputum persisted for several days, often accompanied by tachypnea or tachycardia due to airway infection, air pollution, or other factors, leading to a local or systemic inflammatory response requiring intervention [2]. Hospitalization was defined as any emergency admission excluding scheduled visits.

Categorical variables were compared using the Chi-square test or Fisher's exact test. For continuous variables with a normal distribution, the Student’s t-test was utilized. Multivariate analysis for survival and event-free periods was performed using the Cox proportional hazards regression model. All statistical analyses were conducted using EZR (Easy R for statistical computing; Saitama Medical Center, Jichi Medical University, Saitama, Japan) [10]. Kaplan-Meier curves were generated using Prism version 9.5.0 (GraphPad Software, Boston, MA).

Given the exploratory nature and small sample size of this pilot cohort (n = 22), the multivariable Cox proportional hazards regression models were strictly simplified to avoid overfitting and model instability. Covariates were restricted a priori to key biologically and clinically meaningful confounders, specifically a history of HF, hospitalization, and baseline smoking pack-years. Hazard ratios (HRs) and 95% confidence intervals (CIs) were calculated, and model stability was rigorously monitored. Due to the limited sample size, missing baseline variables were not imputed, and a complete-case analysis was performed.

## Results

Table 1 summarizes the comparison between patients receiving BGF triple therapy in the Department of Pulmonary Medicine and the Department of Cardiology. No significant differences were observed in age, sex, BMI, mortality during follow-up, hospitalization rate, or acute exacerbation rate between the two groups. However, the cardiology group exhibited a higher prevalence of HF and a higher rate of newly initiated BGF therapy.

**Table 1:** Clinical background Continued: Continued medication; Convenience: Prescribed owing to the convenience of use; Escalation: Treatment escalation; Start: Starting the medication. Data in parentheses indicate percentages (%) for categorical variables and standard deviations (SD) for continuous variables.

Factor		Pulmonary department	Cardiology department	P-value
n (%)		15 (68.2)	7 (31.8)	
Age (SD)		80.47 (10.76)	85.57 (3.15)	0.238
Female (%)		2 (13.3)	0 (0.0)	1
Body mass index (SD)		21.83 (3.80)	23.18 (3.62)	0.442
Home oxygen therapy (%)		2 (13.3)	0 (0.0)	1
Heart failure (%)		4 (26.7)	7 (100.0)	0.004
Prescription status (%)	Continued	10 (66.7)	0 (0.0)	<0.001
	Convenience	2 (13.3)	0 (0.0)	
	Escalation	2 (13.3)	0 (0.0)	
	Start	1 (6.7)	7 (100.0)	
Death (%)		1 (6.7)	0 (0.0)	1
Exacerbation (%)		4 (26.7)	3 (42.9)	0.63
Hospitalization (%)		7 (46.7)	1 (14.3)	0.193

**Figure 1:**
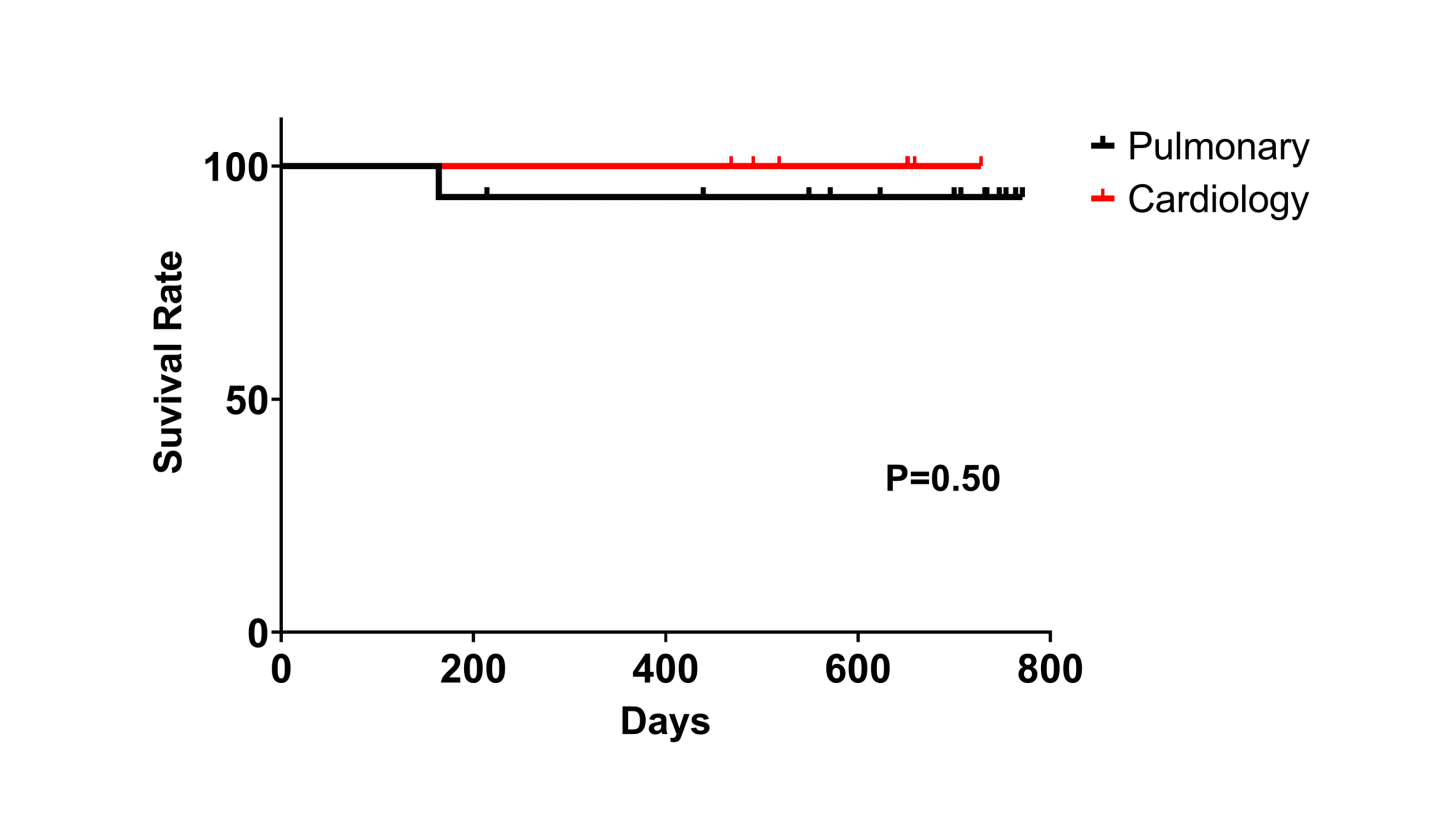
Comparison of overall survival between the cardiology and pulmonary departments Survival rates for patients on budesonide/glycopyrronium/formoterol (BGF) therapy were compared between the managing departments. Log-rank tests showed no significant difference (p > 0.05), suggesting that prognosis under cardiology management is comparable to that under respiratory specialists.

**Figure 2:**
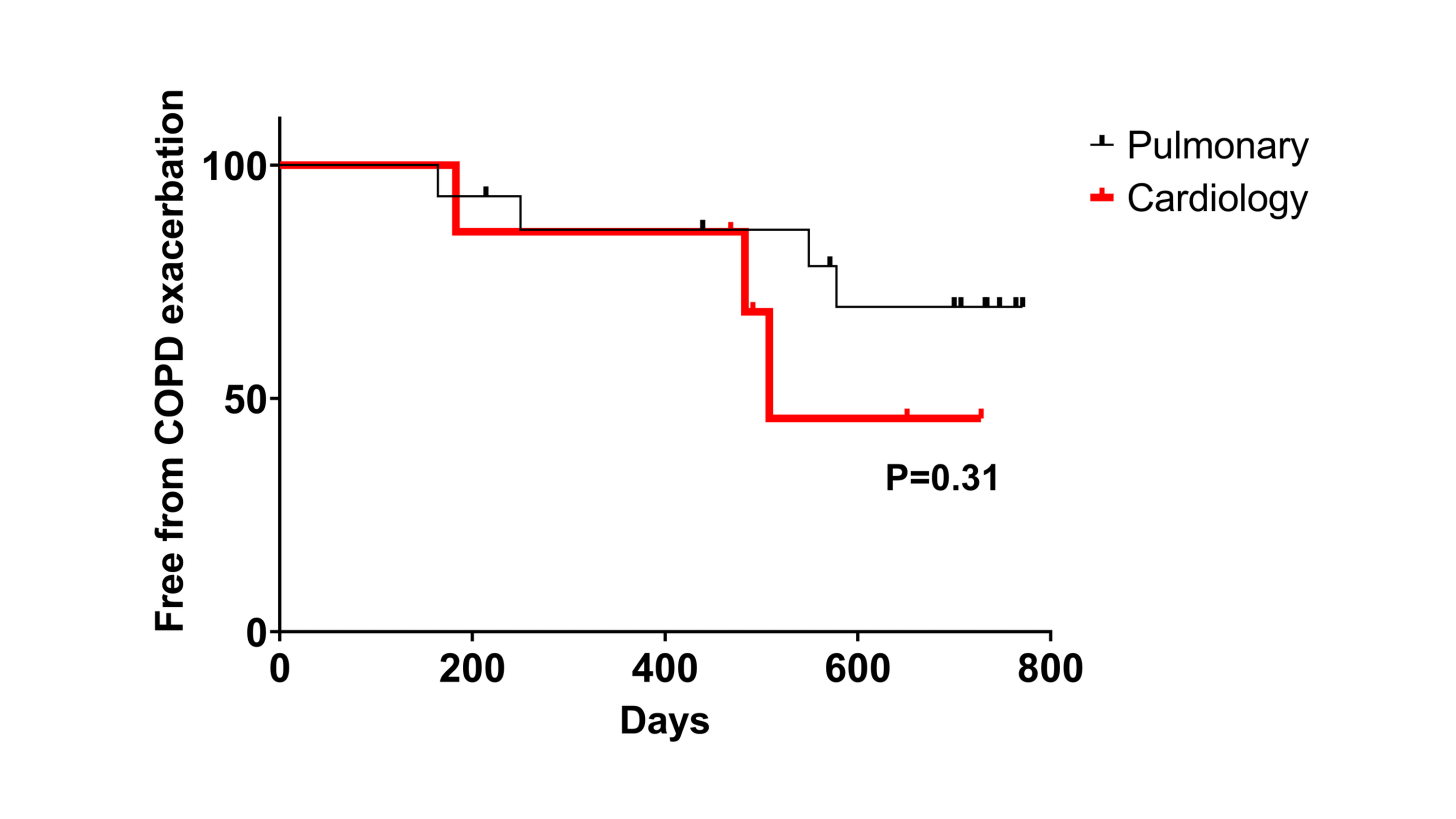
Probability of remaining free from acute exacerbations Kaplan-Meier analysis of the time to the first acute exacerbation after budesonide/glycopyrronium/formoterol (BGF) initiation. Although not statistically significant, a trend toward earlier exacerbation events was observed in the cardiology group, suggesting that background factors, such as heart failure, contribute to risk independently of respiratory symptom severity. COPD: Chronic obstructive pulmonary disease.

**Figure 3:**
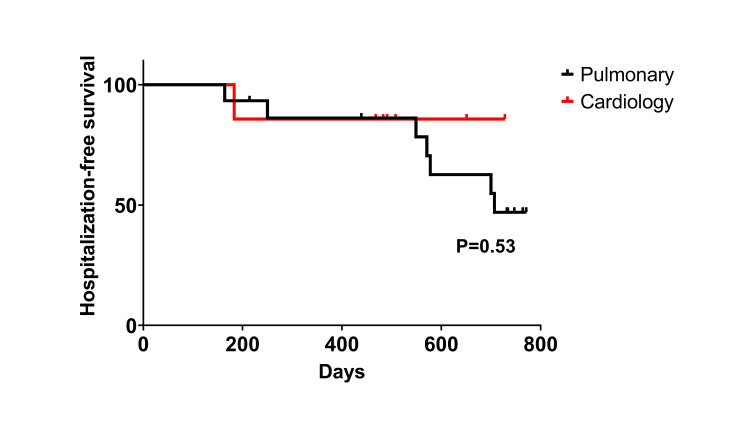
Probability of remaining free from all-cause emergency hospitalizations No significant difference in the emergency hospitalization avoidance rate was observed between the two groups, suggesting that budesonide/glycopyrronium/formoterol (BGF) therapy contributes to the suppression of hospitalizations in clinical practice regardless of the managing department.

Kaplan-Meier curves were used to evaluate overall survival (Figure 1), COPD exacerbation-free probability (Figure 2), and hospitalization-free probability (Figure 3). While no statistically significant differences in event-free probabilities were found between the groups, the cardiology group appeared to have a higher frequency of acute exacerbations.

Subsequently, Cox proportional hazards analysis (Table 2) revealed that BMI and the presence of HF significantly influenced the time to acute exacerbation in patients receiving BGF. A higher BMI was associated with a lower HR for acute exacerbation, whereas comorbid HF significantly increased the HR.

**Table 2:** Cox proportional hazards model HR: Hazard ratio; CI: Confidence interval; AF: Atrial fibrillation; PAF: Paroxysmal atrial fibrillation; Pulmonary: BGF initiation by the Department of Pulmonary Medicine; Convenience: Prescribed owing to the convenience of use; Escalation: Treatment escalation; Start: Starting the medication; Inf: Infinite.

Days to exacerbation	Pre-selection model				Stepwise selection based on P-values			
	HR	95% CI (Lower)	95% CI (Upper)	P-value	HR	95% CI (Lower)	95% CI (Upper)	P-value
Longstanding AF	13.48	0.062	2941	0.34	Excluded by a stepwise procedure			
PAF	0.00000000017	0	Inf	1.00				
Age	0.78	0.46	1.334	0.36				
Body mass index	0.34	0.12	0.97	0.043	0.57	0.33	0.97	0.037
Pulmonary	0.00032	0	Inf	1.00	Excluded by a stepwise procedure			
Male	1018000	0	Inf	1.00				
Heart failure	125200	3.81	4112000000	0.027	212.8	3.32	13650	0.0016
Home oxygen therapy	12.46	0.10	1494	0.30	Excluded by a stepwise procedure			
Convenience	4897	0.57	41840000	0.066				
Escalation	0.0000031	0	Inf	1.00				
Start	0.0012	0	Inf	1.00				

Notably, in this study, there were no cases of infection in the absence of acute exacerbation among patients receiving BGF therapy. Although pre- and post-treatment heart rates were not evaluated in this study, no cases of worsened HF or exacerbated arrhythmias following inhaler initiation were observed in either group.

## Discussion

Our results demonstrated no significant differences in mortality, hospitalization, or acute exacerbation rates between the cardiology and pulmonary groups among patients receiving BGF triple therapy. This finding suggests that, when appropriate diagnosis and guideline-based interventions are implemented, cardiologists can achieve therapeutic outcomes comparable to those of respiratory specialists.

A key finding was that the cardiology group had a significantly higher prevalence of HF and a greater proportion of new BGF initiations. Generally, COPD patients seen in cardiology clinics are often assumed to have milder respiratory impairment than those in specialized respiratory clinics. Consequently, introducing intensive triple therapy like BGF for mild-to-moderate COPD detected "incidentally" during cardiovascular management might be viewed by some as "over-treatment" [11]. However, our data challenge this concern. Multivariate analysis indicated that HF was a significant predictor of acute exacerbation. The tendency for the exacerbation-free curve in the cardiology group to fall below that of the pulmonary group (Figure 2) suggests that even when respiratory symptoms are mild, the presence of HF, a potent comorbidity, requires the patient to be classified as "high risk" within the broader clinical context. Thus, in cases with comorbid HF, the risk of decompensation due to cardiopulmonary interaction may outweigh the significance of pulmonary function metrics alone. This highlights the importance of cardiologists' early and proactive intervention with BGF to prevent exacerbations.

Regarding cost-effectiveness, while medical interventions must be validated economically [12], BGF therapy is superior to other inhalants [13]. Although some literature suggests potential over-treatment [11], these critiques often fail to identify specific ICS formulations or patient phenotypes that are prone to pneumonia. For patients with cardiovascular disease, the benefits of BGF therapy likely outweigh the risks.

The inverse correlation between BMI and the HR for acute exacerbation aligns with the "obesity paradox" in COPD [14-16]. High BMI may contribute to a better prognosis by maintaining nutritional reserves and suppressing systemic inflammation [17,18], whereas sarcopenia may accelerate systemic inflammation [19]. Therefore, strict monitoring and nutritional management may be essential for low-BMI patients, especially those with HF.

However, several caveats warrant caution. The immediate initiation of triple therapy without rigorous, individualized evaluation may be clinically unacceptable in patients with unstable AF or uncompensated HF; management must be strictly tailored to each patient. In patients with borderline cardiovascular stability, a step-up protocol - sequentially introducing long-acting bronchodilators while closely monitoring cardiac rhythm - could serve as a safer alternative to immediate triple therapy. Furthermore, although we used formoterol due to the convenience of the single-inhaler BGF formulation, alternative long-acting beta 2-agonists with a slower onset of action, such as salmeterol, might theoretically minimize transient reflex tachycardia. This remains an important avenue for future comparative investigations.

Several important limitations of this study must be acknowledged. First, this was a single-center, retrospective pilot study with an extremely small sample size (n = 22), which inherently severely limited its statistical power. Consequently, the study was underpowered to demonstrate true clinical equivalence or non-inferiority between cardiologist-led and pulmonologist-led management. Second, significant imbalances in baseline characteristics and the lack of standardized historical parameters, such as structured GOLD staging or objective inhaler adherence scores, introduce unavoidable selection bias and confounding. Third, the wide CIs observed in our initial regression models indicated statistical instability; although we simplified our multivariate models to minimize overfitting, these regression results must be interpreted with extreme caution. Therefore, our findings are not confirmatory but should be positioned as strictly exploratory and hypothesis-generating. Large-scale, prospective, randomized controlled trials are warranted to validate the safety and efficacy of multidisciplinary, collaborative COPD management.

Despite these limitations, healthcare disparities exist, and the gap may widen as advanced devices and drugs are developed [20,21]. This pilot study is significant as it attempts to clarify disparities in COPD care across different clinical departments.

## Conclusions

BGF triple therapy can be safely and effectively administered in the Department of Cardiology. Our findings suggest that for patients with comorbid HF, intensive multifaceted treatment, including BGF, should be considered from the perspective of cardiopulmonary protection, regardless of the severity of respiratory symptoms. Cardiologist-initiated BGF triple therapy is a safe, valid, and essential strategy for mitigating the high risk of acute exacerbations driven by HF, even in patients with seemingly mild respiratory symptoms.
